# A rapid response magnitude scale for timely assessment of the high frequency seismic radiation

**DOI:** 10.1038/s41598-018-26938-9

**Published:** 2018-06-04

**Authors:** M. Picozzi, D. Bindi, D. Spallarossa, D. Di Giacomo, A. Zollo

**Affiliations:** 1 University of Naples Federico II, Naples, Italy; 2 Helmholtz Centre Potsdam, GFZ German Research Centre for Geosciences, Potsdam, Germany; 3 University of Genova, Genova, Italy; 4 International Seismological Centre ISC, Thatcham, United Kingdom

## Abstract

In this work the scaling of seismic moment (M_0_) and radiated energy (E_r_) is investigated for almost 800 earthquakes of the 2016–17 Amatrice-Norcia sequences in Italy, ranging in moment magnitude (M_w_) from 2.5 to 6.5. The analysis of the M_0_-to-E_r_ scaling highlights a breaking of the source self-similarity, with higher stress drops for larger events. Our results show the limitation of using M_0_, and in turn M_w_, to capture the variability of the high frequency ground motion. Since the observed seismicity does not agree with the assumptions on stress drop in the definition of M_w_, we exploit the availability of both E_r_ and M_0_ to modify the definition of M_w_ and introduce a rapid response magnitude (M_r_), which accounts for the dynamic properties of rupture. The new M_r_ scale allows us to improve the prediction of the earthquake shaking potential, as shown by the reduction of the between-event residuals computed for the peak ground velocity. The procedure we propose is therefore a significant step towards a quick assessment of earthquakes damage potential and timely implementation of emergency plans.

## Introduction

In the aftermath of an earthquake, the rapid assessment of both location and extension of potentially damaging ground shaking is a primary task for seismological agencies supporting emergency managers. In this context, shaking maps^1^ become a de-facto standard for a timely dissemination of the ground shaking experienced in the area struck by an earthquake. To improve the spatial resolution of such maps for rapid response actions, the rapid determination of an earthquake size and location supports the information provided by the actual ground motion measurements (if available) and predicted ones. The moment magnitude M_w_^2,3^ is used by the seismological community as the primary measure of the earthquake size. Since M_w_ is based on an estimate of the seismic moment M_0_^4,5^, it provides fault-averaged, low-frequency information on source processes but relatively less information about the small-wavelength high-frequency rupture details^6^. For example, earthquakes with similar M_w_ but different stress drop (Δσ) can generate different ground motion levels^7^, suggesting that a rapid assessment of Δσ could allow more reliable predictions of the earthquake-induced ground motion severity to engineering structures (hereinafter, shaking potential). Moreover, recent studies^8–10^ showed that the Δσ variability is a key parameter for explaining the between-event residuals at short periods. Since different magnitude scales provide different information about the static and dynamic features of the earthquake rupture, magnitudes other than M_w_ could better characterize the earthquake size in terms of high-frequency energy release^11–13^. For example, M_w_ can be complemented with a magnitude scale based on the high-frequency level of the Fourier spectrum^14^. Using teleseismic broadband P-wave recordings, a magnitude scale (M_e_) was introduced^15^ based on measurements of the radiated energy (E_r_) and revising the Gutenberg and Richter relationship^16^ between E_r_ and the surface-wave magnitude M_s_. Since M_e_ is directly linked to the source dynamics, it is more sensitive to high-frequency source details such as variations of the slip and/or stress conditions, and the dynamic friction at the fault surface during the rupture process. Indeed, high energy-to-moment ratios indicate that the intensity of radiated energy at high frequencies is large relative to the size (measured by moment) of an earthquake, with significant implications on hazard assessment^17^. Although automatic procedures for the rapid estimation of M_e_ using P-wave recordings have been proposed both at teleseismic^18^ and local^19^ distances, a strategy to combine the information provided by M_w_ and M_e_ for a rapid assessment of the damage potential of an earthquake has not been proposed yet.

In this study, we present a new procedure to measure the earthquake size using rapid assessments of both E_r_ and M_0_, considering S-wave recordings within 100 km from the epicenter. The methodology is applied to Central Italy, used as an example region where the seismic hazard for residential buildings is dominated by close-distance earthquakes of low-to-moderate magnitude^20^ (i.e., from 4.5 to 6.5). We first calibrate empirical attenuation relationships between the integral of squared velocity (IV2_S_) and the peak displacement (PD_S_) with respect to E_r_ and M_0_, respectively, considering 229 earthquakes ranging from M_w_ 2.4 to 6.1, mostly belonging to the L’Aquila (2009) seismic sequence^21^. Then, the procedure is applied to about 775 earthquakes of the 2016–2017 Central Italy sequence^22^ with M_w_ in the range 2.5–6.5. Innovatively, we use the estimates of E_r_ and M_0_ (i.e., through the slowness parameter^23^) to introduce a rapid response magnitude (M_r_). The new M_r_ magnitude scale is tied to the non-saturating M_w_, but it includes a term which accounts for the difference between the earthquake dynamic conditions and those assumed by Kanamori for the original definition of M_w_. Finally, the impact of the new magnitude scale is discussed in terms of assessment of the earthquake shaking potential quantified through the peak ground velocity (PGV) and peak ground acceleration (PGA).

## Background

The moment magnitude M_w_ is defined as^2^

1 $${{\rm{M}}}_{{\rm{w}}}=({{\rm{l}}{\rm{o}}{\rm{g}}{\rm{M}}}_{0}{\textstyle \text{-}}9.1)/1.5=({{\rm{l}}{\rm{o}}{\rm{g}}{\rm{M}}}_{0}{\textstyle \text{-}}4.3{\textstyle \text{-}}4.8)/1.5,$$

where the slope value of 1.5 and the constant −4.8 are inherited from the relationship between log(E_r_) and the surface-wave magnitude M_s_^16^, whilst the term −4.3 derives from the following assumptions: (a) the energy required for fracturing is negligible; (b) the final average stress and the average stress during faulting are equal (also known as “complete stress drop” Δσ or “Orowan’s model”^24^); (c) the average rigidity in the source area (μ) is ranging from 3 to 6 × 10^4^ MPa under average crust-upper mantle conditions; (d) Δσ is nearly constant for very large earthquakes, with values between 2 and 6 MPa.

The above assumptions can be also formalized as:

2 $${{\rm{E}}}_{{\rm{r}}}/{{\rm{M}}}_{{\rm{0}}}={\rm{\Delta }}{\rm{\sigma }}/(2{\rm{\mu }})=5\times {10}^{-5}$$

Introducing the slowness parameter term θ = log(E_r_/M_0_)^23^, equation (2) corresponds to:

3 $${{\rm{\theta }}}_{{\rm{K}}}=-\,4.3$$

that is, θ assumes the value −4.3 under the Kanamori assumptions^2^ on Δσ and μ. However, it is well-known that rupture processes may deviate from the complete stress drop, ranging between the “partial stress drop”^25^ and “frictional overshoot”^26^ models. Global datasets of earthquakes with M_w_ varying between 5.5 and 9 suggest that θ varies between −4.7 and −4.9^15,^^27,28^, which would correspond to an average global stress drop a factor 3 to 4 smaller than that assumed by Kanamori^2^. This difference in the average stress drop values is also reflected by the expected over estimation of about 0.27 m.u. of M_e_ with respect to M_w_ when an earthquake satisfies the condition θ = θ_K_^29^.

### Dataset

In this study, we analyze about 1000 earthquakes with M_w_ between 2.5 and 6.5, occurred in Central Italy between July 2008 and September 2017 (see Text Sa). This area of the Apennines is characterized by southwest-northeast extension (i.e., active NE- and SW-dipping normal and normal-oblique faults) since the Middle Pliocene^30^.

**Figure 1:**
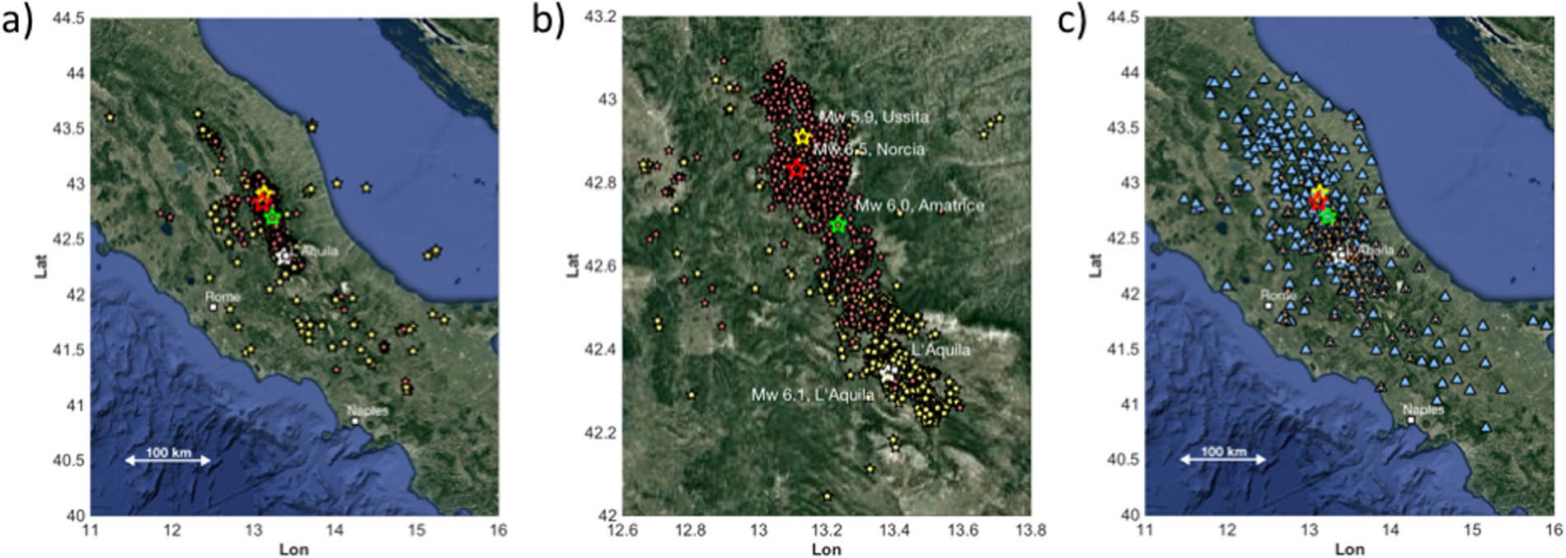
(**a**) Locations of the earthquakes considered in this study, occurring between 2009 and 2015 (yellow) and between 2016 and 2017 (red); (**b**) zoom in of the epicentral area, with the locations of the largest earthquakes shown by stars. (**c**) Location of the seismic stations relevant to either the calibration (orange reverse triangles) or application (cyan triangles) datasets. Maps were made using MATLAB (R2016b; 9.1.0.441655; https://it.mathworks.com/, last accessed January 2018).

The dataset includes the main seismic sequences that have occurred over the past 10 years in Central Italy: the 2009 L’Aquila sequence^21^, and the 2016–2017 Central Italy sequence^31^ (Fig. 1a). The four largest analyzed earthquakes (M_w_ ≥ 5.9) are: 1) the April 6, 2009 M_w_ 6.3, which occurred near the town of L’Aquila and caused about 300 fatalities, 2) the August 24, 2016 M_w_ 6.0, Amatrice earthquake, 3) the October 26, 2016 M_w_ 5.9 Visso earthquake, and 3) the October 30, 2015 M_w_ 6.5 Norcia earthquake, which ruptured a ~40 km long fault, bridging the seismic gap left from the previous two earthquakes^22^.

The data were recorded by the Italian strong motion network (RAN) and by the permanent and temporary stations of other networks (see Text Sa and Fig. 1). To reproduce a rapid response procedure, the recordings are pre-processed and the squared-velocity parameter (IV2_S_) and the peak displacement (PD_S_) are both measured on direct S-waves following an analysis scheme suitable for real-time operations^32^, (see Text Sb). From the whole compiled dataset, which consists of more than 1400 earthquakes, we extracted a subset considering the following selections: hypocentral distance smaller than 100 km; events recorded by a minimum of 8 stations and stations having at least 8 records; the sum of SNR for the three components ≥200. The selected dataset is composed by 1004 earthquakes recorded at 340 stations. This dataset is split in two parts. The first part consists of 229 earthquakes, which occurred between July 2008 and January 2016 (hereinafter referred to as dataset D1), including the 2009 L’Aquila M_w_ 6.3 sequence. Data set D1 is used to calibrate the attenuation models used in this study. The second dataset (hereinafter referred to as D2) is composed of 775 earthquakes occurred in the study area since January 2016 and it is used to exemplify the application of the proposed methodologies. For D2, the number of good quality recordings per event varies from 10 to 50 (Figure S1).

## Results

### Rapid response assessment of radiated energy and seismic moment

The rapid assessment of E_r_ and M_0_ is based on extracting, from the time histories, proxies for these two source parameters, and correcting them for attenuation along the path. In particular, the radiated seismic energy is estimated from the squared velocity integrated over the S-wave time window^33^ (IV2_S_), whereas the seismic moment is derived from the S-wave peak-displacement^34^ (PD_S_). The parameters IV2_S_ and PD_S_ are linked to E_r_ and M_0_ through empirical attenuation models derived using data set D1, as detailed in the section Method (eqs 8 and 9). Since linear non-parametric regressions^19^ are applied, the attenuation models are tables listing the attenuation coefficients for the discretized distance range. The attenuation models are reported in Table S1.

The attenuation models (8) and (9) calibrated over data set D1 are used to compute E_r_ and M_0_ of earthquakes included in D2. The E_r_ and M_0_ values are obtained by averaging over several recording stations (see Text Sc and Figure S2 for details).

**Figure 2:**
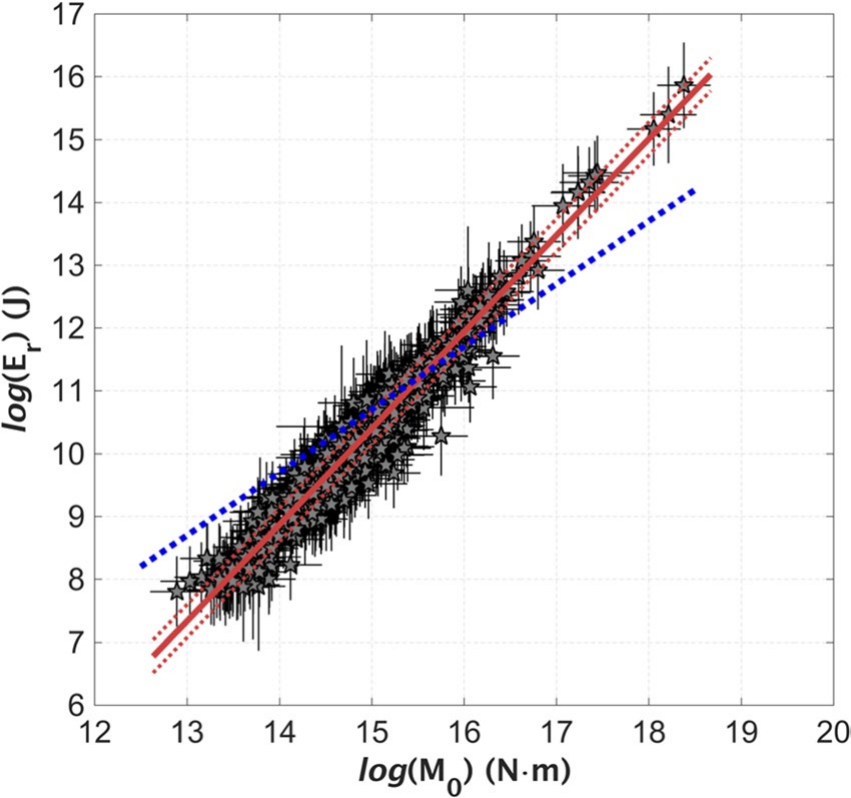
E_r_ versus M_0_ computed for the 2016–2017 sequence (grey stars, with ±1 standard deviations shown as vertical and horizontal bars); the best-fit model (mean ±1 standard deviation are shown as red lines) is compared to the energy-to-moment ratio assumed by Kanamori^2^ (blue dashed line).

Figure (2) shows that a linear scaling between E_r_ and M_0_ holds over 7 orders of magnitude for seismic moment. The observed M_0_-to-E_r_ scaling well compares to that of other tectonic areas (Figure S3). The best-fit model is:

4 $$\mathrm{log}({{\rm{E}}}_{{\rm{r}}})=(1{\rm{.53}}\pm {\rm{0}}\mathrm{.02})\mathrm{log}({{\rm{M}}}_{{\rm{0}}})-(\mathrm{12}{\rm{.59}}\pm {\rm{0.26}})$$

with a coefficient of determination (R^2^) equal to 0.94 and a standard deviation equal to 0.26.

The model of Eq. (4) deviates from the M_0_-to-E_r_ scaling related to the Kanamori’s condition (i.e., log (E_r_) = log (M_0_) + θ_K_), shown in Fig. (2) as blue dashed line. The obtained slope larger than 1 (equation 4) implies a breaking of the source self-similarity, with stress drops larger for larger events. Moreover, the value of 12.59 obtained for the intercept implies that the condition θ = θ_K_ is met by the analyzed data set for M_0_ = 10^15.58^ [Nm], corresponding to M_w_ = 4.3, as shown in Fig. (2).

### Energy to moment scaling and event specific adjustment to M_w_

A straightforward application for rapid response purposes of the E_r_ and M_0_ estimates derived for the 2016–2017 dataset would consist of converting them into energy and moment magnitudes. In the case of M_0_, we adopt the original definition proposed by Kanamori^2^ (i.e., Eq. 1). By contrast, in the case of the energy magnitude, we use the calibration dataset to parameterize a linear relationship between E_r_ and the local magnitude (M_L_)^35^. The best-fit model obtained by an iteratively reweighted least squares^36^ and with 200 bootstrap replications^37^ considered to assess the uncertainties (Figure S4) is:

5 $${{\rm{M}}}_{{\rm{L}}}=(0.568\pm 0.01)\mathrm{log}({{\rm{E}}}_{{\rm{r}}})-(2.54\pm 0.1);$$

with R^2^ equal to 0.93 and the standard deviation of the residuals is equal to 0.13. Applying Eq. (5), the derived magnitudes agree with M_L_, but being tied to E_r_, can be extended toward larger earthquakes to avoid magnitude saturation effects^38^. The magnitudes obtained by the 2016–2017 E_r_ estimates are referred to as energy-based local magnitude^19^ (M_le_).

**Figure 3:**
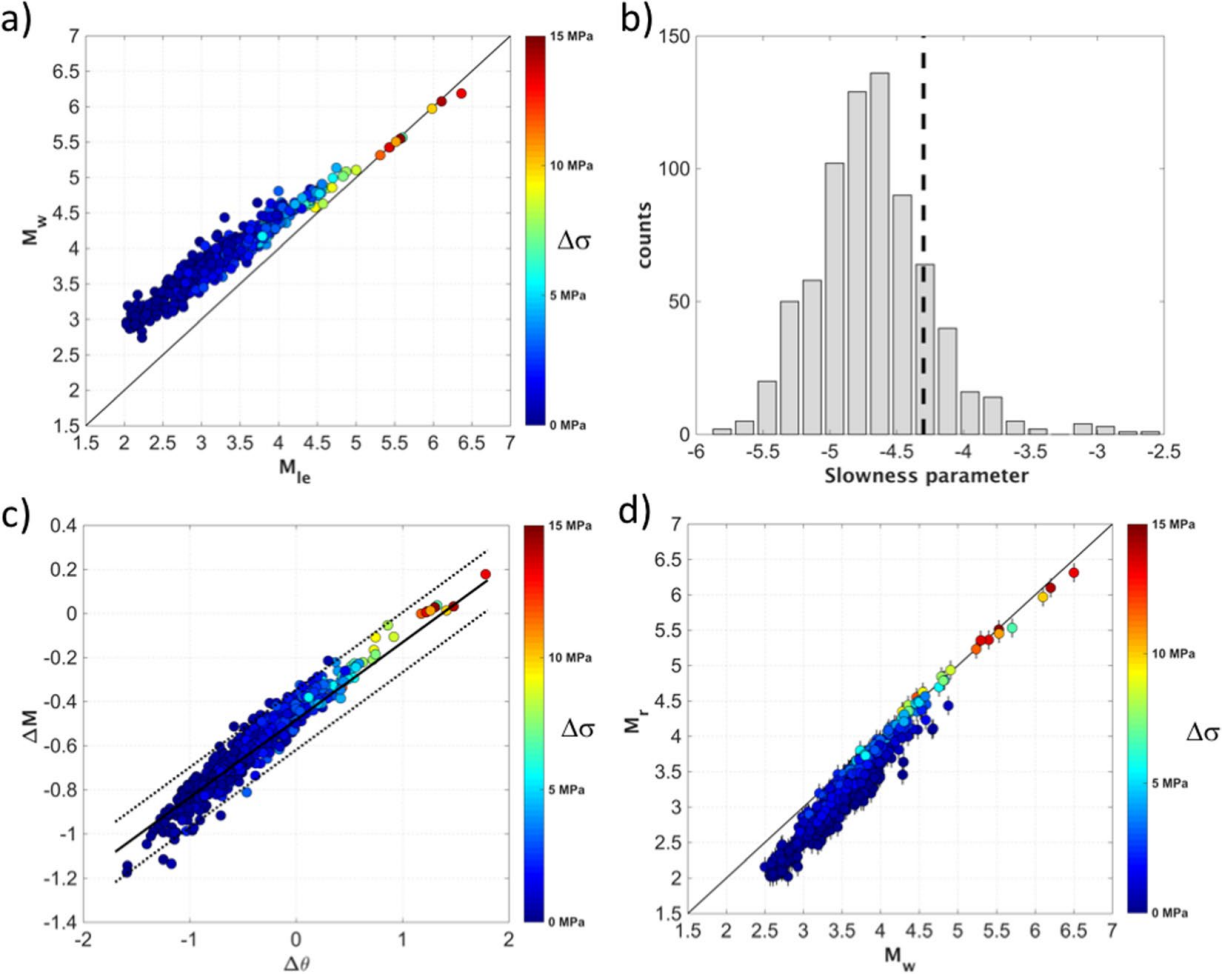
(**a**) Moment magnitude (M_w_) versus energy-based local magnitude (M_le_), colored per Δσ. (**b**) Distribution of the slowness ratio θ(grey) and θ_K_ = −4.3 (black dashed line). (**c**) Distribution of Δθ = θ − θ_K_ versus ΔM = (M_le_ − M_w_) (circles) compared to the best-fit model given in Eq. 8 (the mean and the mean ±1 standard deviation are the continuous and dashed black lines, respectively). (**d**) Rapid response magnitude (M_r_) versus M_w_ circles, with ±1 standard deviation bars); the 1:1 relationship (black) is shown for reference.

Figure (3a) shows that, while M_w_ and M_le_ well agree for M_w_ > 5, for smaller events M_w_ tends to progressively overestimate M_le_, and the (M_w_ − M_le_) difference increases with decreasing stress-drop (Δσ)^39^.

The M_0_-to-E_r_ scaling (Fig. 2) and the differences between the energy and moment magnitudes in relation to Δσ (Fig. 3a) reflect a regional behavior of the source scaling which deviates from the Kanamori’s assumptions θ_K_ = −4.3 (Eq. 3). As shown in Fig. (2), the ratio log(E_r_/M_0_) is expected to be smaller than the values implied by θ_K_ = −4.3 for M_w_ < 4.3. Considering the magnitude distribution of the analyzed earthquakes, most of the empirical θ values are smaller than θ_K_ Fig. 3b, in agreement with Δσ smaller than the value assumed by Kanamori^2^. Therefore, these earthquakes can potentially generate a ground shaking variability at high frequency larger than the variability expected by assuming θ constant as in Eq. (1).

In the context of early warning and rapid response applications where event-specific analysis is of concern, it is worth considering a new magnitude scale that accounts for the differences in the events dynamic conditions with respect to those assumed by Kanamori for the original definition of M_w_.

Figure (3c) shows that ΔM = (M_le_ − M_w_) scales with Δθ = (θ − θ_K_) and correlate with Δσ While Δθ provides a mean for inferring, in the rapid response timeframe, how much the occurring earthquake deviates from the Kanamori’s condition (i.e., if the earthquake is as energetic as it is expected from the θ_K_ condition), an empirical linear model between Δθ and ΔM can be used for modifying the original definition of M_w_:

6 $${\rm{\Delta }}M=(0.34\pm 0.04){\rm{\Delta }}{\rm{\theta }}\,-(0.48\pm 0.04);$$

with R^2^ equal to 0.88 and a standard deviation, assessed by a bootstrap approach^37^, equal to 0.16.

Therefore, given Eq. (6), ΔM can be added to M_w_ for deriving a new rapid response magnitude scale:

7 $${{\rm{M}}}_{{\rm{r}}}={\rm{\Delta }}M+(\mathrm{log}\,{{\rm{M}}}_{{\rm{0}}}-{\rm{9.1}})/1{\rm{.5}}={\rm{\Delta }}M+{{\rm{M}}}_{{\rm{w}}}$$

where M_r_ is used in the remainder of this work to indicate the new magnitude scale introduced to take into account the event-specific deviation of θ from θ_K_. Figure (3d) compares M_r_ with M_w_ estimated by applying a non-parametric spectral inversion approach^39^. It is worth noting that for earthquakes characterized by Δσ values from ~5 to ~15 MPa, ΔM varies between −0.2 to 0.2 magnitude units. Therefore, the difference between M_r_ and M_w_ is negligible for the largest events of the sequence (i.e. for M_w_ > 5) which fulfill Kanamori’s condition. However, for smaller earthquakes characterized by Δσ below 2 MPa, the application of the ΔM factor leads to M_r_ being significantly smaller than M_w_ (Fig. 3d).

To quantify the impact of M_r_ on predicting the ground shaking potential, we follow the earthquake engineering approach of analyzing the event-specific deviations from the expected mean regional attenuation trend. We refer to PGV as a measure of earthquake shaking potential, since it is controlled by the seismic energy at intermediate frequencies and, hence, better suited than peak ground acceleration (PGA) for statistical analysis correlating ground motion with damage^40^. We calibrate a ground motion prediction equation (GMPE) to model the PGV scaling with distance and magnitude in the target area (see Text Sc). The calibration is repeated twice, considering either M_w_ or M_r_. Then, the between-event residuals^41^ (δBe) are computed as the average difference, for any given event, between the PGV measured at different stations and the corresponding values predicted by the GMPE. However, considering that PGA can be anyway of interest for engineering^42^ or seismological^10,43,44^ applications, the same analyses are repeated for PGA, (see Text Sc).

**Figure 4:**
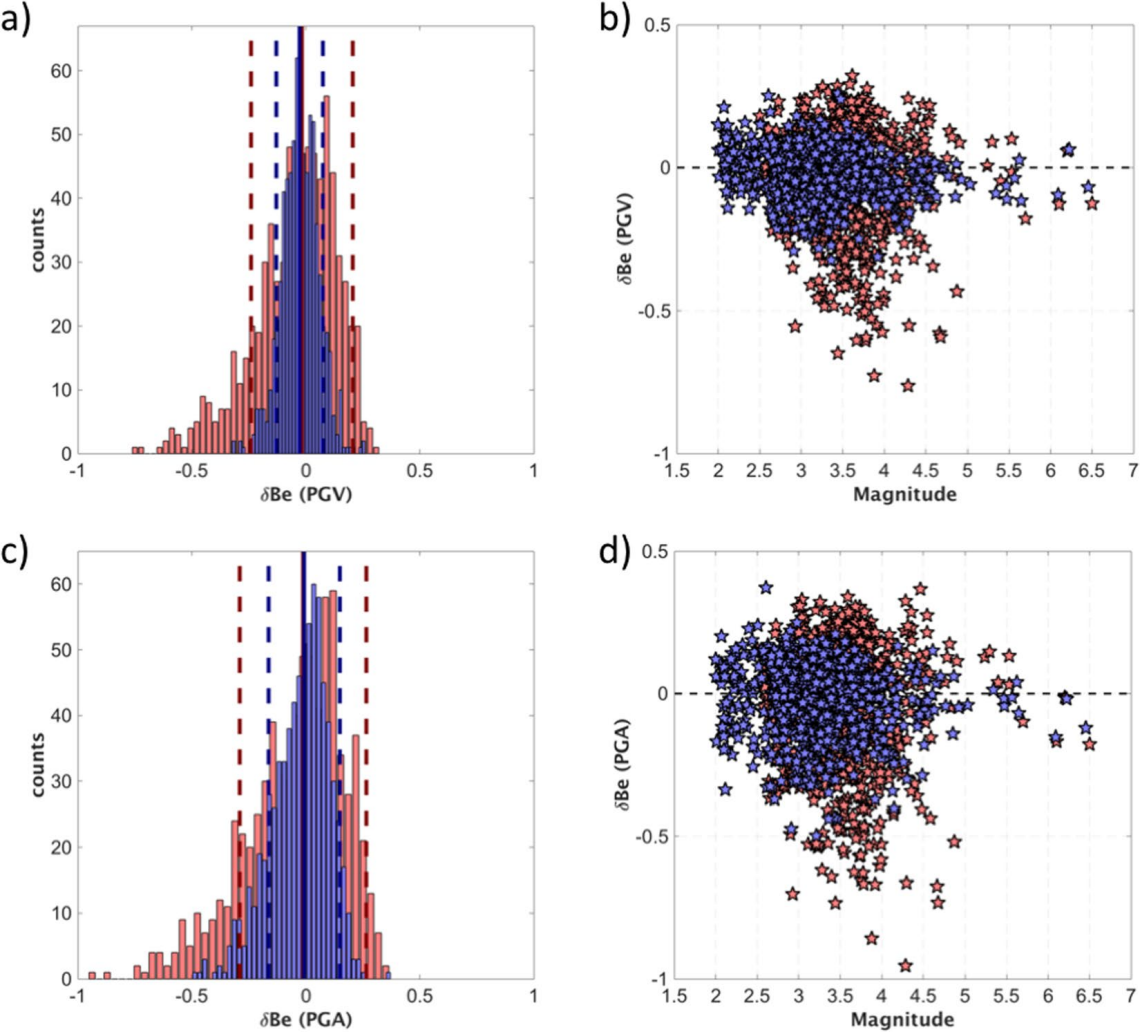
(**a**) Histograms of the between-event residuals (δBe) computed for PGV considering either M_w_ (red) or M_r_ (blue); the ±1 standard deviations are red and blue dashed lines for M_w_ and M_r_, respectively. (**b**) δBe residuals for PGV considering either M_w_ (red) or M_r_ (blue) versus M_w_; the zero-bias value (dashed line) is shown for reference. (**c**) Same as a), but δBe computed for PGA. (**d**) Same as b), but δBe computed for PGA.

Figure (4a) shows that the standard deviation of δBe computed considering PGV reduces from 0.18 to 0.08 when M_w_ is replaced with M_r_, meaning that M_r_ better accounts for those variations in the rupture process which can introduce systematic event-dependent deviations from the mean regional PGV scaling. As shown in Fig. (4b), the reduced δBe variability is mostly related to the small magnitude events (i.e. M_w_ < 4.5) which show the largest deviation from the θ = θ_K_ condition. A similar result is obtained also for PGA (Fig. 4c and d), for which the standard deviation of δBe reduces from 0.22 to 0.13 when M_w_ is replaced by M_r_.

## Discussion

This study was motivated by the need of developing a procedure for the rapid assessment of the earthquake shaking potential. To accomplish such a task, we worked on two topics: first, we introduced an approach for the rapid assessment of the seismic moment and the radiated energy; second, we used these two source parameters to define a measure of the earthquake size which is not only informative for the average tectonic effects but it accounts for the efficiency of the earthquake to radiate seismic energy at high-frequency.

Concerning the first topic, standard approaches adopted by seismic observatories for estimating M_0_ from moment tensor analysis^45,46^ are delayed by the requirement of acquiring full waveforms at regional-teleseismic distances. For instance, in Italy the moment tensor solutions are provided by INGV generally between 6 to 9 minutes after the earthquake occurrence (http://info.terremoti.ingv.it/en/help#TDMT) and ShakeMaps are released to the Civil Protection after this time span (http://shakemap.rm.ingv.it/shake/about.html). In this work, we have proposed an approach for quick and robust estimations of M_0_ and E_r_ based on proxies measured on S-waves recorded by a local network. The application of the attenuation models calibrated for both M_0_ and E_r_ to 775 earthquakes of the 2016–2017 Central Italy sequence confirmed the suitability of the approach for rapid-response applications.

Figure (S8) shows the comparison between the E_r_ estimates obtained by the procedure proposed in this study, which follows Picozzi *et al*.^19^, and the E_r_ obtained for the same dataset by a non-parametric inversion approach^39^, which follows Kanamori *et al*.^38^. As shown within Figure (S8), the two considered procedures provide consistent results, within a factor 1.5/2 (i.e., the log(E_r_) differences are within 0.2/0.3 log_10_ units). We observe that the rapid estimates of E_r_ tend to be slightly larger than those computed by Bindi *et al*.^39^. At least part of this overestimation could be ascribed to local site amplification effects, which have been considered in Bindi *et al*.^39^ through site correction terms, not included in equation (8), an issue of interest for future studies. However, considering that E_r_ estimates on the same earthquakes provided by different investigators often differ up to one order of magnitude^47^, the result supports the reliability of the rapid estimates of E_r_ from IV2_S_. The availability of a dense network in the area under study was certainly an important condition to average out radiation pattern and rupture directivity effects on E_r_ and M_0_ estimates. Future examination will explore the bias on E_r_ and M_0_ estimates for network configurations less dense than the one considered.

Regarding the earthquake damage potential assessment, the analyzed data set represented a suitable test-case to discuss the limitation of using M_0_ to capture the variability of the high frequency ground motion. The topic is so important that it has been recently debated by different studies^7–10^. In this study, we have considered the same scientific topic but from the point of view of an observatory which must provide real-time information to support civil protection decisions and seismic risk mitigation actions. It is important to note that early-warning and rapid-response systems often deal with seismic sequences occurring in a specific target region. Since the within-sequence stress drop variability is expected to be larger than the between-mainshock stress drop variability^48^, the E_r_-to- M_0_ scaling can deviate from the assumptions behind the definition of M_w_^2^. For example, Δσ of the earthquakes analyzed in this study varies from about 0.1 to 15 MPa, leading to slowness ratios significantly different from θ_K_ (Fig. 3b).

The rapid assessment of M_0_ and E_r_ can thus be exploited to define a new rapid response magnitude scale (M_r_) tied to M_w_ but including a term that accounts for the difference between the actual energy-to-moment ratio and the value used by Kanamori^2^.

To demonstrate the potential value of the rapid response magnitude M_r_ in better capturing the event-to-event shaking potential variability, we quantified the reduction of the between-event standard deviation for PGV and PGA, which is controlled by the Δσ variability. Considering Δσ values^37^ and the scheme proposed by Choy^49^ over the energy-to-moment ratio, we observe that earthquakes with M_w_ > 4 (showing Δσ from 5 to 15 MPa) are moderately or highly energetic, whilst small magnitude events (characterized by Δσ < 1 MPa) are enervated (Figure S5). In our opinion, such large variability in the source properties justifies the introduction of the new magnitude scale for rapid response procedures, which is capable of better predicting the ground motion over a frequency range of engineering interest.

The proposed approach for computing E_r_, M_0_ and M_r_ can be implemented into algorithms for real-time operations^50,51^, so that M_r_ estimates can be available as soon as the S-waves are recorded by a minimum number of stations that allow stable E_r_ and M_0_ estimates to be obtained (i.e., within a few tens of seconds, depending on the seismic network density and telemetry efficiency), and can be exported to other areas for which the attenuation model for E_r_ and M_0_ can be calibrated. The application of the procedure for magnitudes larger than those in our dataset might be influenced by the saturation limit of the proxies used for the estimation of E_r_ and M_0_. We expect that extending the observation scale from local to regional networks and considering other seismic phases than direct S-waves would allow for estimates of M_r_ to be extended to earthquakes with magnitude larger than M_w_ 6.5. The saturation limit for the rapid-response procedure here proposed will be investigated in future studies.

The implications of our results are for a broad scientific audience (e.g., seismological, engineering seismology, disaster manager communities). Indeed, the implementation of our procedure for computing the seismic moment (M_0_), the radiated energy (E_r_) and M_r_ in real-time operations can allow civil protection operators to better assess within a rapid response timeframe (i.e., within a few tens of seconds from the earthquake’s origin time) an earthquake damage potential and the timely implementation of emergency plans. Furthermore, considering that M_r_ reduces the variability of the between-event residuals^41^ with respect to M_w_, we foresee its possible application in the development of ground motion prediction equations for seismic hazard assessment.

## Method

### Calibration of the empirical relationships IV2_S_-E_r_ and PD_S_-M_0_

Following previous studies^19^, we have derived an empirical relationship between IV2_S_ and E_r_. To this purpose, we considered 229 earthquakes, and we computed the theoretical seismic radiated energy following a procedure^52^ based on the spectral integration of the squared theoretical Brune’s velocity spectrum for S-waves^25^. These have been derived using the corner frequency (f_c_) and seismic moment (M_0_) computed in previous studies^8,53^.

Hence, we related the IV2_S_ estimated for each recording to E_r_ radiated by the source assuming a linear model, where the attenuation of average IV2_S_ amplitude as a function of distance is expressed without assuming any a-priori functional form (i.e., non-parametric, or data-driven, approach):

8 $$log[IV{2}_{S}({R}_{H})]=A+Blog({E}_{r})+{w}_{j}{C}_{j}+(1-{w}_{j}){C}_{j+1}$$

where the hypocentral distance R_H_ range is discretized into N_bin_; the index *j* = 1,…, N_bin_ indicates the *j-th* node selected such that R_H_ is between the distances r_j_ ≤ R_H_ < r_j+1_; the attenuation function is linearized between nodes r_j_ and r_j+1_ using the weights *w*, computed as *w*_j_ = (r_j+1_ − R_H_)/(r_j+1_ − r_j_).

**Figure 5:**
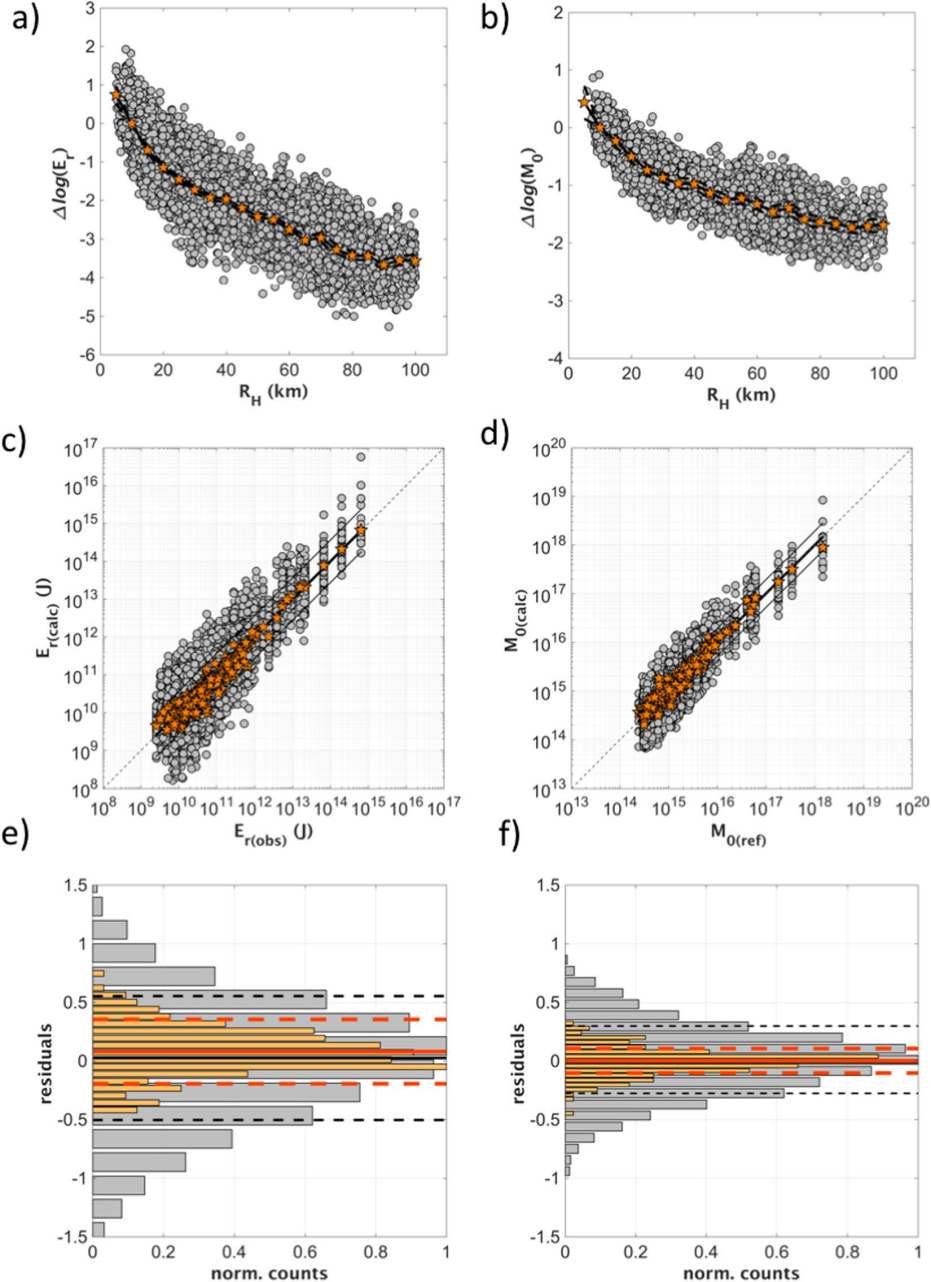
Results of the calibrations between IV2_S_ and E_r_ (Eq. 8) and between PD_S_ and M_0_ (Eq. 9). (**a**) The coefficients C_j_ of Eq. 8 (orange) are compared with the residuals $${\rm{\Delta }}log({E}_{r})=log[IV{2}_{S}({R}_{H})]-A-Blog({E}_{r})$$ (grey circles); (**b**) the coefficients G_j_ in Eq. 9 (orange circles) are compared with the residuals $$\Delta log({M}_{0})=log$$$$[P{D}_{S}({R}_{H})]-D+Flog({M}_{0})$$ (grey circles); (**c**) the IV2_S_ values corrected for C_j_ are compared with the energy E_r(obs)_ (the corrected values for each recording are in grey, the average for each earthquake in orange); (**d**) PD_S_ values corrected for G_j_ are compared with M_0(obs)_ (the corrected values for each recording are in grey, the average for each earthquake in orange); (**e**) histograms of the residual distribution computed for Eq. (8) considering either single recording (grey, with ±1 standard deviation, dashed black lines) or the average computed by grouping recordings per event (orange). (**f**) The same as in panel e), but considering Eq. (9).

The R_H_ range 5–100 km is discretized into 19 bins with equal width (i.e., 5 km) (Fig. 5a). The minimum distance is fixed to 5 km given the lack of recordings at shorter distances. The coefficients A, B, C_j_ are determined by solving the over-determined linear system (8) in a least-square sense. To fix the trade-off between A and C_j_, the attenuation is constrained to zero at r_2_ = 10 km (i.e., at the upper boundary of the first distance bin). However, it is worth noting that changing the node constrained to zero corresponds to changes of both the A and C_j_ coefficients which compensate each other, so that the coefficient B in Eq. (8) is unaltered.

Similarly, the relationship between the peak displacement PD_S_ and M_0_ has been expressed in the form:

9 $$log[P{D}_{S}({R}_{H})]=D+Flog({M}_{0})+{w}_{j}{G}_{j}+(1-{w}_{j}){G}_{j+1}$$

The coefficients A, B, ${C}_{j}$ and D, F, ${G}_{j}$ are reported in Table S1. The calculated regressions have a R^2^ equal to 0.84 and 0.87, respectively.

The scaling with distance of the derived attenuation models is shown in Fig. 5a and b, where the distance distribution of the observed IV2_S_ and PD_S_ values corrected for the source scaling A + B log(E_r_) and D + F log(M_0_) are compared with the attenuation models ${C}_{j}$ and ${G}_{j}$, respectively (i.e., where the index *j* indicates different bins of hypocentral distances). For both cases, the good agreement with the corrected data confirms the suitability of the obtained attenuation models.

Figure 5c and d compare the observed IV2_S_ and PD_S_ values corrected for attenuation effects, as modelled through the ${C}_{j}$ and ${G}_{j}$ coefficients, with E_r_ and M_0_. The source scaling (black line) defined by the coefficients A, B and D, F capture well the trend in the data over the entire energy and moment ranges, as indicated by the agreement with the median value obtained for each event by averaging over more than 10 recording (yellow circles in Fig. 5c and d). Finally, Fig. 5e and f summarize the residual distributions between predicted and observed log(E_r_) and log(M_0_). Considering the values computed for each recording (gray), the residuals are unbiased with standard deviations equals to 0.56 and 0.29 for E_r_ and M_0_, respectively; when the average values per event are considered (yellow), the standard deviations reduce to 0.27 and 0.1, respectively.

### Data and Resources

The waveforms used in this study have been obtained from European Integrated Data Archive-EIDA (https://www.orfeus-eu.org/data/eida/) and from the Italian Civil Protection (DPC) repository (http://ran.protezionecivile.it/IT/index.php). Regarding the permanent networks, we used data from the networks with FDSN (http://www.fdsn.org/networks/) code: MN (10.13127/SD/fBBBtDtd6q), IV (10.13127/SD/X0FXnH7QfY), IT (10.7914/SN/IT). All of the webpages were last visited in March 2018. The analysis has been performed using the MATLAB software (R2016b; 9.1.0.441655; https://it.mathworks.com/, last accessed January 2018). Data generated during this study are available from the corresponding author on request.

## Electronic supplementary material

Supplementary Information
