# Supplementary material for: A rapid response magnitude scale for timely assessment of the high frequency seismic radiation
Source: Sci Rep. 2018 Jun 4;8:8562. doi: 10.1038/s41598-018-26938-9 (PMC5986871; doi:10.1038/s41598-018-26938-9)
Supplement: Supplementary file 1 — Supplementary Information [file 41598_2018_26938_MOESM1_ESM.doc]

**A rapid response magnitude scale for timely assessment of the high frequency seismic radiation**

Picozzi M.^1^*, Bindi D.^2^, D. Spallarossa^3^, D. Di Giacomo^4^, and A. Zollo^1^

^1^University of Naples Federico II, Italy

^2^Helmholtz Centre Potsdam, GFZ German Research Centre for Geosciences, Germany

^3^University of Genova, Italy

^4^International Seismological Centre ISC, United Kingdom

  *Corresponding author: matteo.picozzi@unina.it

**Introduction**

The Supplementary Information contains information about:

1. Description of the dataset
2. Data processing
3. Calibration of a Ground Motion Prediction Equation for the peak ground velocity (PGV) and peak ground acceleration (PGA) considering M_w_ and M_r_
4. Regression parameters of Eq. (8) and Eq. (9)
5. Description of the dataset

The dataset considered in this study consists of about 200000 waveforms relevant to 1004 earthquakes occurred in Central Italy between 2008 and 2017, and recorded at 340 stations. The dataset includes all the largest earthquakes occurred in Central Italy in the past 10 years (i.e., the M_w_ 6.3 L’Aquila, M_w_ 6.2 Amatrice, M_w_ 6.1 Ussita, and M_w_ 6.5 Norcia earthquakes). The data consists of both velocimetric and accelerometric recordings for earthquakes spanning the magnitude range from 2.5 to 6.5 and hypocentral distances < 150 km. 75% of the hypocentral depths are shallower than 10km. The considered networks are: The National Seismic Network (RSN), the Mediterranean Network (Mednet), the Rapid Response Networks operated by the Istituto Nazionale di Geofisica e Vulcanologia (INGV), and the National Accelerometric Network (RAN) operated by the Department of Civil Protection (DPC), (see Data and Resources).

1. Data processing

This section presents the data processing for computing the peak displacement (PD_S_) and the cumulative squared velocity (IV2_S_) for direct S-waves. Preliminary waveforms selection and processing were carried out as follows: automatic phase identification using the Rsni-Picker^1,2^ (i.e., the picker in operation at the Regional Seismic network of North-western Italy); visual inspection of the original waveform to validate P and S onsets and to identify and reject signals clipped or contaminated by transient noise; Butterworth band pass pre-deconvolution filter with a variable high pass corner frequency in the range between 0.05 and 0.4 Hz determined by signal-to-noise ratio (SNR) analysis (i.e., this latter made on accelerometric data). A low pass corner frequency was fixed to 40 Hz. For accelerometric data recorded by the RAN network, we adopted the same processing procedure of the Italian strong-motion database ITACA^3,4^; instrumental correction and computation of acceleration, velocity and displacement. The parameters PD_S_ and IV2_S_^5,6^ are computed considering a time window starting 0.1 s before the S-wave onset and ending at different percentages of the total energy as a function of the source to site distance R: (i) 90 per cent when R < 25 km; (ii) 80 per cent when 25 km < R < 50 km; (iii) 70 per cent when R > 50 km. For both PD_S_ and IV2_S_ calculations, we imposed a minimum time window length of 5 s and a maximum time window length of 20 s. For each recording, a signal-to-noise ratio was evaluated considering a pre-event noise window of the same length as the signal window. Finally, the values of the three components of ground motion are summed for both PD_S_ and IV2_S_.

From the whole compiled dataset, we extracted a subset considering the following selections: hypocentral distance smaller than 100 km; events recorded by a minimum of 8 stations and stations having at least 8 records; the sum of SNR for the three components ≥ 200.

1. **Calibration of a Ground Motion Prediction Equation for the peak ground velocity (PGV) and peak ground acceleration (PGA) considering M_w_ and M_r_**

To compute the between-event residuals^7^ (δBe) for peak ground velocity (PGV), and considering that most of the events in our dataset have magnitude below the minimum magnitude for which ground motion prediction equations (GMPE) have been calibrated^8^ for the area (i.e., M_w_ < 4), we performed a multivariate linear regression analysis to search the best-fit parameters of the equations

log(PGV) = A_V_ + B1_V_*(M_w_-Mref) + B2_V_*(M_w_-Mref)^2^ + C_V_*log(R), (Eq. S1)

and

log(PGV) = A’_V_ + B1’_V_*(M_r_-Mref) + B2’_V_*(M_r_-Mref)^2^ + C’_V_*log(R), (Eq. S2)

where R is the hypocentral distance in km and PGV is in cm⋅s^-1^, and the reference magnitude Mref is set equal to 3.5 (Figure S5).

The magnitude range is from M_w_ 2.5 to M_w_ 6.5 (i.e., red bars in Figure S6), the hypocentral distances span from 5 km to 100 km. The regression analyses provide: for Eq. (S1), A_V_ = 0.81, B1_V_ = 1.12, B2_V_ = -0.05 and C_V_ = -1.66, with a standard error of 0.576; for Eq. (2), A’_V_ =1.08, B1’_V_ = 0.93, B2’_V_ = -0.015, and C’_V_ = -1.68, with a standard error of 0.55.

Figures S7a and S7b show the PGV residuals (i.e., observed versus theoretical) as a function of the hypocentral distance and magnitude, respectively.

A similar analysis was carried out also for the peak ground acceleration (PGA). Also in this case, we performed a multivariate linear regression analysis to search the best-fit parameters of the equations

log(PGA) = A_A_ + B1_A_*(M_w_-Mref) + B2_A_*(M_w_-Mref)^2^ + C_A_*log(R), (Eq. S3)

and

log(PGA) = A’_A_ + B1’_A_*(M_r_-Mref) + B2’_A_*(M_r_-Mref)^2^ + C’_A_*log(R), (Eq. S4)

The regression analyses provide: for Eq. (S3), A_A_ = 2.71, B1_A_ = 0.88, B2_A_ = -0.01 and C_A_ = -1.90, with a standard error of 0.41; for Eq. (S4), A’_A_ =2.95, B1’_A_ = 0.77, B2’_V_ = -1.6·10^-4^, and C’_V_ = -1.93, with a standard error of 0.37.

Figures S7c and S7d shows the PGA residuals (i.e., observed versus theoretical) as a function of the hypocentral distance and magnitude, respectively.

**Figures**


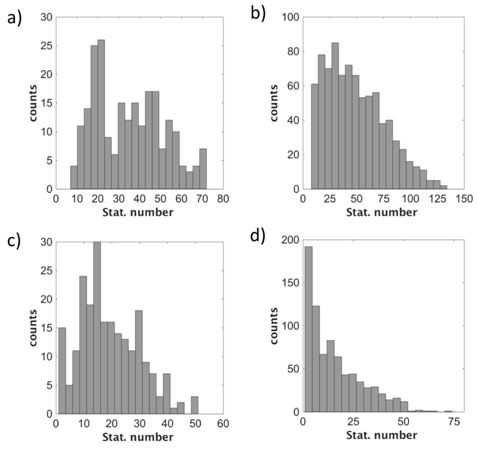


**Supplementary Figure S1:** a) Number of recordings for individual events in the calibration dataset used for the parameterization of the PD_S_ versus M_0_ model. b) Same as a), but for the IV2_S_ versus E_r_ model. c) Number of recordings in the 2016-2017 dataset for the estimation of M_0_. d) Same as c), but for the estimation of E_r_.


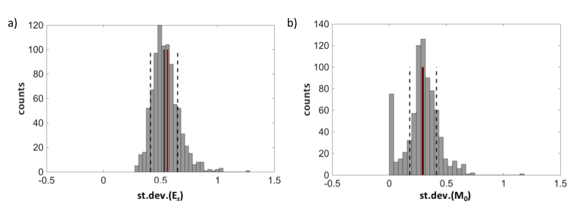


**Supplementary Figure S2:** a) Distribution of standard deviation for E_r_ computed for individual event recordings (gray), median (black line), 16^th^ and 84^th^ percentiles (black dashed line), standard deviation from Eq. (4). b) Same as a), but for M_0_.


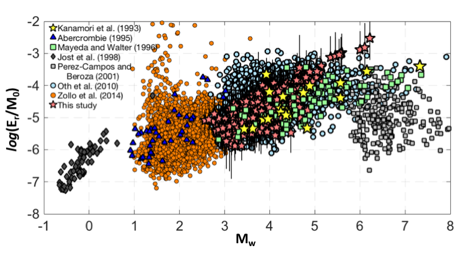


**Supplementary Figure S3**: Scaled energy versus moment magnitude for the dataset analyzed in the present study (red stars) and seven additional other seismological data sets from tectonic areas worldwide involving a wide range of distances (i.e., from local to teleseismic) and seismic phases (e.g., direct P- and S-waves and coda)^8-10^.


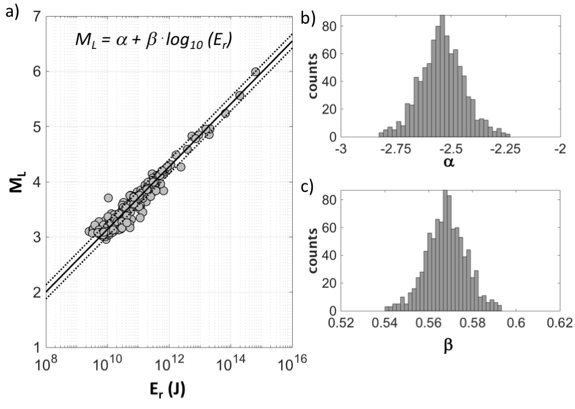


**Supplementary Figure S4:** a) M_L_ versus E_r_, best-fit line (black) and ± 1 standard deviation (black dashed lines). b) and c) distribution of the α and β parameters in the E_r_ versus M_L_ linear relationship, respectively.


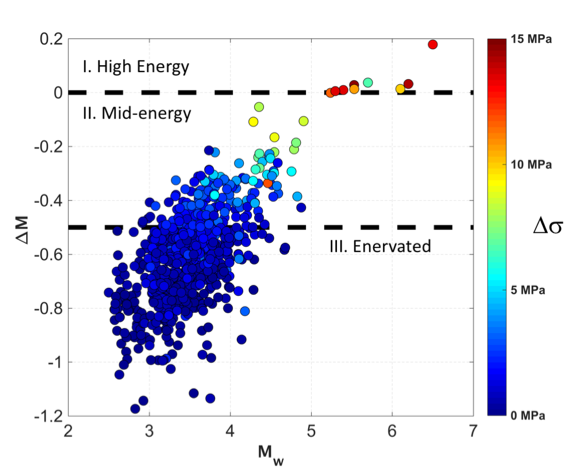


**Supplementary Figure S5:** Difference between local-energy magnitude (M_le_) and moment magnitude (M_w_) with respect to M_w_), colored per Δσ. Limits for the discrimination between Enervated, Mid-Energy, and High Energy events as proposed by Choy^11^ (black dashed lines).


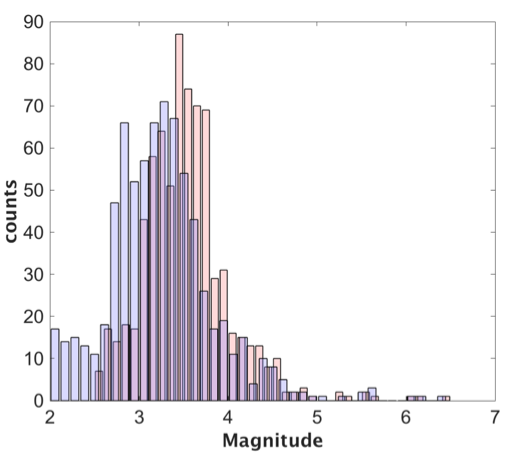


**Supplementary Figure S6:** Magnitude distributions for the 2016-2017 Central Italy dataset, M_w_ (red), M_r_ (light blue).


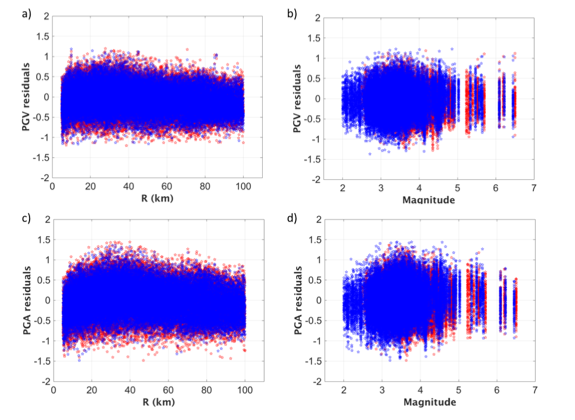


**Supplementary Figure S7:** a) PGV residuals from Eq. (S1) (red) and Eq. (S2) (blue) of the Supplementary Information versus hypocentral distance. b) Same as a), but for magnitude. c) Same as a), but for PGA. d) Same as b), but for PGA.


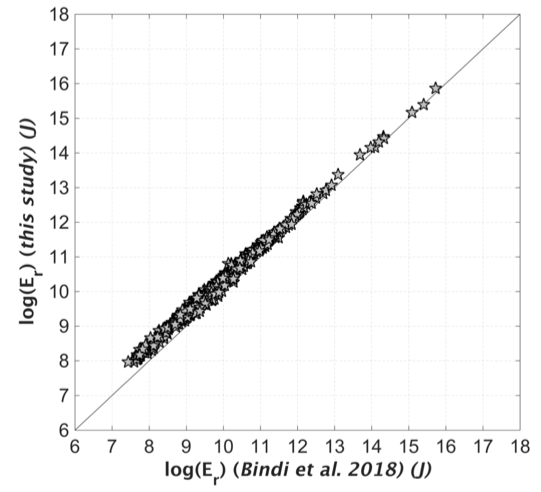


Supplementary Figure S8: E_r_ computed for the 2016-2017 sequence by a non-parametric inversion approach by Bindi et al.^12^ versus E_r_ values computed in this study on the same data (grey stars); the 1:1 relationship (black) is shown for reference.

1. **R**egression parameters of Eq. (8) and Eq. (9)

| **Parameters** | **Mean** ± **Stand. dev.** | | **Parameters** | **Mean** ± **Stand. dev.** | |
| --- | --- | --- | --- | --- | --- |
| **A** | -12.52 ± 0.08 | | **D** | -16.91 ± 0.11 | |
| **B** | 1.052 ± 0.007 | | **F** | 0.986 ± 0.007 | |
|  |  | |  |  | |
| **Parameters** | **Values** | **Stand. dev.** | **Parameters** | **Values** | **Stand. dev.** |
| **C_1_** | 0.746 | 0.114 | **G_1_** | 0.439 | 0.146 |
| **C_2_** | 0.0 | 0.0 | **G_2_** | 0.0 | 0.0 |
| **C_3_** | -0.693 | 0.045 | **G_3_** | -0.233 | 0.050 |
| **C_4_** | -1.147 | 0.038 | **G_4_** | -0.503 | 0.046 |
| **C_5_** | -1.450 | 0.038 | **G_5_** | -0.739 | 0.047 |
| **C_6_** | -1.723 | 0.042 | **G_6_** | -0.870 | 0.044 |
| **C_7_** | -1.927 | 0.039 | **G_7_** | -0.969 | 0.045 |
| **C_8_** | -1.965 | 0.043 | **G_8_** | -0.981 | 0.046 |
| **C_9_** | -2.197 | 0.049 | **G_9_** | -1.138 | 0.047 |
| **C_10_** | -2.417 | 0.050 | **G_10_** | -1.266 | 0.046 |
| **C_11_** | -2.494 | 0.047 | **G_11_** | -1.229 | 0.048 |
| **C_12_** | -2.757 | 0.047 | **G_12_** | -1.332 | 0.047 |
| **C_13_** | -3.031 | 0.053 | **G_13_** | -1.465 | 0.052 |
| **C_14_** | -2.965 | 0.065 | **G_14_** | -1.401 | 0.054 |
| **C_15_** | -3.277 | 0.056 | **G_15_** | -1.560 | 0.051 |
| **C_16_** | -3.425 | 0.055 | **G_16_** | -1.640 | 0.047 |
| **C_17_** | -3.437 | 0.059 | **G_17_** | -1.670 | 0.052 |
| **C_18_** | -3.669 | 0.067 | **G_18_** | -1.733 | 0.048 |
| **C_19_** | -3.553 | 0.064 | **G_19_** | -1.708 | 0.056 |
| **C_20_** | -3.567 | 0.078 | **G_20_** | -1.689 | 0.055 |

**Supplementary Table S1:** Regression parameters of Eq. (8) and Eq. (9) with their standard deviations for the calibration dataset.

**References**

1. Spallarossa, D., *et al.* Performance of the RSNI- Picker. *Seismol. Res. Lett.* **85**, 6, 1243–1254. doi: 10.1785/0220130136. (2014).
2. Scafidi, D., *et al.* Automatic P‐ and S‐Wave Local Earthquake Tomography: Testing Performance of the Automatic Phase‐Picker Engine RSNI‐Picker. *Bull. Seism. Soc. Am.* **106**, 2, 526–536. https://doi.org/10.1785/0120150084. (2016).
3. Luzi, L., *et al.* ITACA (ITalian ACcelerometric Archive): A web portal for the dissemination of Italian strong motion data. *Seismol. Res. Lett.* **79**, 5, doi:10.1785/gssrl.79.5. (2008).
4. Paolucci, R., *et al.* Record processing in ITACA, the new Italian strong motion database. In *Earthquake Data in Engineering Seismology, Geotechnical, Geological and Earthquake Engineering Series* **14**, pp. 99–113, eds Akkar, S., Gulkan, P. & Van Eck, T., Springer, Berlin. Kanamori et. al., 1993. (2011).
5. Brondi, P., *et al.* Predicting the macroseismic intensity from early radiated P wave energy for on-site earthquake early warning in Italy. *J. Geophys. Res. Solid Earth* **120**, 7174–7189, doi:10.1002/2015JB012367. (2015).
6. Al Atik L., *et al.* The Variability of Ground-Motion Prediction Models and Its Components. *Seismological Research Letters* **81**, 5, p. 794-801, doi:10.1785/gssrl.81.5.794. (2010).
7. Bindi, D., *et al.* Ground motion prediction equations derived from the Italian strong motion database. *Bull. Earthq. Eng.* **9**, 6, 1899–1920. (2011).
8. Oth, A., *et al.* Earthquake scaling characteristics and the scale-(in)dependence of seismic energy-to-moment ratio: Insights from KiK-net data in Japan. *Geophys. Res. Lett.* **37**, L19304, doi:10.1029/2010GL044572. (2010).
9. Ide, S., & Beroza G. C. (2001), Does apparent stress vary with earthquake size? *Geophys. Res. Lett.* **28**, 3349-3352. (2001).
10. Zollo, A., *et al.* Source parameter scaling and radiation efficiency of microearthquakes along the Irpinia fault zone in southern Apennines, Italy. *J. Geophys. Res. Solid Earth* **119**, 3256–3275, doi:10.1002/2013JB010116. (2014).
11. Choy, G.L. Stress conditions inferable from modern magnitudes: development of a model of fault maturity. **IS3.5** *in Bormann, P. (Ed.). New Manual of Seismological Observatory Practice (NMSOP-2), IASPEI, GFZ Potsdam*, http://nmsop.gfz-potsdam.de; DOI: 10.2312/GFZ.NMSOP-2. (2012).
12. Bindi D., Spallarossa, D., Pacor, F., Picozzi, M., Scafidi, D., & Weatherill, G. Impact of magnitude selection on aleatory variability associated with Ground Motion Prediction Equations: Part I – local, energy and moment magnitude calibration for Central Italy, Accepted for publication in Bull. Seismol. Soc. Am. doi: 10.1785/0120170356. (2018).
